# The effectiveness of a brief video-based intervention in reducing gender bias in Korea

**DOI:** 10.3389/fpsyg.2024.1331460

**Published:** 2024-04-09

**Authors:** Yejin Bae, Jisun Jeong

**Affiliations:** ^1^Department of Counseling Psychology Education, Graduate School of Education, Korea University, Seoul, Republic of Korea; ^2^Department of Education, Pusan National University, Busan, Republic of Korea

**Keywords:** Video Interventions for Diversity in STEM, VIDS, perspective taking, gender groups, gender equity in Korea

## Abstract

**Introduction:**

Gender bias deepens gender disparities by fueling gender conflicts. Thus, effective interventions for gender bias are necessary. Understanding gender discrimination experienced by another gender, both emotionally and logically, may contribute to reducing gender bias in Korean society. Hence, we conducted an online experiment using Video Interventions for Diversity in Science, Technology, Engineering, and Mathematics (VIDS) to examine the effectiveness of shortened VIDS intervention through perspective taking in reducing gender bias.

**Methods:**

A sample of Korean adults aged 19–39 (*n* = 160, 61.8% women, 38.2% men) were recruited. In the treatment group, male participants watched VIDS videos that portrayed a woman getting gender prejudiced and female participants watched VIDS videos showing a man receiving gender biased treatment in the society. The videos presented to treatment group consisted of one narrative and one expert video from VIDS, which stimulate emotional and logical understanding of the another gender, respectively. Participants in the control group watched a control video that was irrelevant to gender bias. All participants then answered gender bias questionnaire, as well as cultural orientation questionnaire.

**Results:**

Cultural orientation as a covariance, ANCOVA (Analysis of Covariance) revealed participants in the treatment group showed significantly lower gender bias than the control group. Within the treatment group, a moderation analysis showed that logical thinking moderated the relationship between emotional immersion and decreased gender bias, meaning stimulated logical thinking from watching the videos affected how engaging emotionally to the another gender’s situation lowers gender bias.

**Discussion:**

Our findings suggest that VIDS, a video-based gender bias intervention tool, can still be effective when edited briefly. Furthermore, one’s perspective-taking strategy can be considered when trying to decrease gender bias through videos that promote perspective-taking. The findings highlight the possibility of utilizing short video intervention that enhances perspective taking on decreasing gender bias.

## Introduction

The issue of gender equity has emerged significant worldwide and within Korea, necessitating immediate action. Compared to Korea’s rapid economic growth over the past several decades the progress towards gender equity has been slow, fueled by gender conflicts stemming from gender biases within the society. More than 50% of men and women aged 19–34, claim that their own gender is more discriminated against within the Korean society (The Ministry of Gender Equality and Family, 2021). This perception gap is significant in the early 20s, specifically in the ages 19–24 attributed to their experience from adolescence to adulthood (The Ministry of Gender Equality and Family, 2021). Although, having received relatively unprejudiced treatment in schools, families, etc., young adults currently encounter pre-existing gender biases in society. Therefore, to combat gender bias and pursue gender equity in Korea, understanding each gender’s perspective and reducing gender biases towards the younger generations, is significant.

The current study designed an online experiment utilizing verified video-based gender bias intervention, VIDS (Video Interventions for Diversity in Science, Technology, Engineering, and Mathematics STEM). Developed to decrease gender bias and raise awareness of gender stereotypes that exist in STEM (Science, Technology, Engineering and Math), VIDS has emerged as an effective video intervention in previous studies (Pietri et al., 2017; Hennes et al., 2018; Moss-Racusin et al., 2018; Pietri et al., 2019). However, the length of the VIDS videos utilized in previous studies is rarely brief. Additionally, these studies have been majorly conducted in Western cultures, especially in the United States. Thus, this study addresses the possibility of using a shortened version of the VIDS to effectively reduce gender bias in non-western Korean society.

To promote perspective-taking between the genders, we presented VIDS videos depicting reversed scenarios of current gender biases that they hold against each other; Men tend to view women as less competent than themselves, and women perceive men to be more assertive (Hentschel et al., 2019). Following this, we examined the effect of the intervention through Analysis of Covariance (ANCOVA), with cultural orientation as the covariance. Additionally, we analyzed how different types of VIDS videos would promote different aspects of perspective-taking (emotional immersion and logical thinking) and explored how the effectiveness of each video interacts in reducing gender bias through moderation analysis. The findings of the experiment could provide a time-saving way to decrease gender bias by promoting the understanding of various gender life experiences, as well as offer possibilities to expand the use of VIDS to the general public in eastern countries.

To this end, the current study first presents the theoretical background on gender bias, its characteristics in Korea, and perspective-taking. The next part of the paper covers material and methods, including the procedure of the online experiment. Lastly, the results, discussion, limitations and future directions, and conclusion are presented.

### Objectives

The current study aimed to a. investigate the effectiveness of shortened VIDS videos on reducing gender bias within Korean participants and b. Explore the interaction between two dimensions of perspective-taking (emotional immersion and logical thinking) on decreasing gender bias. We assessed and controlled cultural orientation to aim for an unbiased assessment of the effectiveness of the intervention. Further, moderation analysis was employed to investigate the impact of emotional immersion and logical thinking on bias reduction.

### Hypotheses

The current research suggests two main hypotheses: Hypothesis 1; Under controlled cultural orientation, individuals in the treatment group would report reduced gender bias after watching the shortened VIDS intervention compared to the control group and Hypothesis 2; In the treatment group, the level of increased emotional engagement and logical thinking from watching the narrative and expert video would show different levels of effectiveness in reducing gender bias.

## Literature review

### Gender bias

Gender bias is the act of showing preferences towards one gender over another (Cook, 2016). Gender bias often enables developing a quick perception of a person based on gender, and various factors such as experience, traditions, principles, values, and culture can contribute to the formation of gender bias (International Labour Organization, 2017). Previous research has shown that negative and prevalent gender biases may exacerbate existing gender disparities (Casad et al., 2020). Korea is notable for its gender disparities, with the highest gender payment gap among Organization for Economic Co-operation and Development (OECD) countries (Organisation for Economic Co-operation and Development, 2017). Female workers in South Korea earn 63% of the wages of their male counterparts. Furthermore, women hold only 17% of seats in the Korean National Assembly, which is the fifth lowest among OECD countries. Existing gender disparities are worsened by gender stereotypes; Gender bias could aggravate discrimination in hiring and decrease opportunities at work, often frustrating women’s goals to advance their careers (Casad et al., 2020). Therefore, efforts to reduce gender stereotypes in Korean society would help in reducing gender disparity and promoting gender equality.

Additionally, existing gender biases within society prevent both genders in their efforts to achieve gender parity. The Ministry of Gender Equality and Family and the Korean Women’s Development Institute (The Ministry of Gender Equality and Family, 2021) stated that the younger generation in Korea, aged 19–34 years, experiences gender discrimination differently. Specifically, 74.6% of women answered that society discriminates against them, whereas according to 51.7% of men society was more discriminating against men, and the tendency to perceive one’s own gender more discriminated is larger for participants in their early 20s. The Ministry of Gender Equality and Family (2021) asserted that the perception gap stems from having to conform to existing gender biases and gender roles as adults, even though their parents and teachers set equal expectations while growing up. Similarly, European countries experience little gender gap in education, but gender gap within society is still observed (Gaweł and Krstić, 2021). Persistent and repeated gender biases in society have widened the gap in perceived discrimination between genders. This gap hinders both genders from openly discussing their experiences of discrimination and making efforts to promote gender equality (Kim, 2019). Moreover, people believe in an arguments’ validity if they become familiar with it through repetition (DeMarzo et al., 2003). Thus, minimizing the repetition of gender biases could contribute to achieving gender parity.

Gender discrimination often occurs online, and more than 50% of men and women encounter sexist content in online communities (The Ministry of Gender Equality and Family, 2021). This can be attributed to the anonymous nature of the Internet, which allows its users to perform acts that they would normally not in identifiable settings (Fox et al., 2015). Thus, we presume that conducting an experiment to decrease gender bias in anonymous online communities would be beneficial. Thus, this study recruited participants from anonymous online communities and conducted an online experiment to understand the efficiency of VIDS in reducing gender bias through perspective taking.

### Perspective-taking

Perspective-taking is the ability to understand how a situation is perceived by others and their cognitive and emotional ways of coping (Gehlbach, 2004). Perspective taking is commonly defined based on two dimensions: Affective and cognitive. Affective perspective-taking emphasizes emotion and can be described as the capacity to the emotions or feelings of another individual. In contrast, Cognitive perspective taking can be characterized as the capacity to understand the beliefs or thoughts of another individual (Healey and Grossman, 2018). Thus, both emotional and logical engagement with others is important in perspective taking. Therefore, gaining an understanding of another gender’s experience emotionally and logically may promote perspective-taking, which could be effective as a gender-biased intervention. Perspective-taking promotes willingness to communicate with an outgroup (Wang et al., 2014. p. 5), reduces intergroup bias (Hutchings et al., 2021), and decreases stereotype actions towards the outgroup (Shih et al., 2009). Moreover, perspective-taking increases intergroup harmony without neglecting existing intergroup disparities and helps lower intergroup bias in society (Todd and Galinsky, 2014; Hutchings et al., 2021). Therefore, perspective-taking may help build a foundation for understanding different groups. Considering that young adults in Korea perceive their own gender as the most discriminated against (The Ministry of Gender Equality and Family, 2021), taking the perspective of another gender could be effective in decreasing gender bias.

For accurate perspective taking, it is important to obtain new and correct information from others (Eyal et al., 2018). Thus, both emotional and logical engagement with others is important in perspective taking. Therefore, gaining an understanding of another gender’s experience emotionally and logically may promote perspective-taking, which could be effective as a gender-biased intervention.

#### Cultural orientation on taking perspective

Understanding other people’s viewpoint relates to how one interacts with the world. Cultural orientation refers to how an individual perceives and acts against the world according to his or her cultural identification, defined by individualism–collectivism and vertical-horizontal dimensions (Singelis et al., 1995; Triandis, 1995). To examine the unbiased impact of perspective-taking on gender bias with video intervention, we first investigated the dimensions of cultural orientation as well as their effect on gender bias and perspective-taking ability.

There are four main dimensions of cultural orientation: horizontal, vertical, and vertical collectivism (Triandis and Gelfand, 1998). People with collectivist cultural traits are likely to identify themselves as part of groups, whereas those in individualist cultures consider themselves as independent (Triandis, 2002). Additionally, people in the vertical dimension recognize themselves within the hierarchy of the in-group, whereas people in the horizontal dimension consider themselves as equal to their in-group members (Soh and Leong, 2002).

Previous research shows that an individual’s cultural orientation influences gender bias (Hang-yue et al., 2006; Harris and Minor, 2020). Specifically, people in collectivist and non-hierarchical cultures have an explicit bias towards women in executive leadership (Harris and Minor, 2020). Cultural orientation can also affect perspective-taking abilities. According to Wu and Keysar (2007), people with collectivist cultural traits (e.g., Chinese) are more inclined to adopt perspectives than people with individualistic cultural traits (e.g., Americans). Thus, people who share a collectivist culture, such as Koreans, may find perspective taking easier than those with different cultural orientations (Lee et al., 2007). Further, of the four cultural orientations, only horizontal collectivism was associated with a greater favor toward altruism which is explained by their motivation to ensure sociability with others and maintain close relationships (Shavitt et al., 2011; Booysen et al., 2021). Considering that altruism and empathy are interrelated, a horizontal collectivist cultural orientation could affect assessments of the effectiveness of an empathy-promoting video intervention (MacNeill and Wozniak, 2018).

In short, having a horizontal collectivist cultural orientation may affect one’s discrimination between genders and result in a greater understanding of others’ perspectives. Thus, in the current study, we aimed to examine the effect of horizontal collectivism along with the other three dimensions of cultural orientation. Our goal was to explore and control the correlation between horizontal collectivism on gender bias to separate the impact of the video intervention, used to promote perspective-taking on gender bias.

#### Using media to promote perspective taking

Interventions for reducing bias are most effective when presented in various forms. Few of the widely used bias interventions include reading a novel, watching a video, or participating in a simulator where the participants encounter discriminatory scenarios (Robinson et al., 2020). Recently, various types of audiovisual media such as video games, videos, and virtual reality devices (Vieira, 2014; Aitamurto et al., 2018; Eyal et al., 2018; Hamilton-Giachritsis et al., 2018; Herrera et al., 2018) have been used to promote perspective-taking.

Videos can be an effective perspective-taking tool because they enhance viewers’ ability to observe a situation, avoid the depersonalization of characters, and provide nonverbal information (Chan et al., 2010). Previous research indicates that video-based education, which includes narratives, promotes empathy, and students think more logically when watching videos in class (Harwood and Mcmahon, 1997; Sweeney and Baker, 2017). Furthermore, people perceive emotion more effectively and accurately when both voice and facial expression are perceived simultaneously (Freeman and Ambady, 2011). Listening to an actual voice results in understanding others better in comparison to reading the same-content text (Schroeder and Epley, 2015). Thus, the current study utilized video as a perspective-taking tool for two main reasons: it is an effective medium for promoting emotional immersion, and an efficient educational tool for increasing logical thinking.

To successfully sustain participants’ attention, the current study utilized videos shorter than six minutes (Guo et al., 2014). Additionally, the total viewing time was limited to less than 11 min because video completion percentages of shorter (less than 11 min) videos were higher among viewers compared to 11 min or longer videos (Geri et al., 2017). Furthermore, adding interactive questions about the video content significantly increased the completion percentage of short (less than 11 min) videos compared to similar videos with no questions presented (Geri et al., 2017). Thus, the current study encouraged participants by providing them attention-check questions to complete the video stimuli and excluded those who displayed less attention.

## Materials and methods

Utilizing an online experiment, this study aimed to explore the efficacy of brief video interventions that promote perspective-taking in decreasing gender bias. For video intervention, we employed the VIDS, developed by Pietri et al. (2017) and utilized by Moss-Racusin et al. (2018) for approximately 10 min to reduce gender bias. VIDS is a series of videos produced to reduce gender bias in STEM. VIDS includes 12 different 5-min-long videos, which can be used under various time constraints; The VIDS consists of six narrative-condition videos that promote empathy and six expert-condition videos that increase logical understanding (Pietri et al., 2017). We used one video from each condition (narrative and expert) to promote two main dimensions of perspective-taking: emotional immersion and logical thinking. Specifically, for male participants, we presented one narrative video and one expert video that portrayed the subtle gender biases that women faced, and one narrative video and one expert video that depicted the subtle gender biases that men faced for female participants. To encourage the participants to watch the videos to the end, all video interventions provided were under 11 min (Geri et al., 2017). Cultural orientation was assessed and controlled for to ensure an unbiased assessment of the efficacy of the shortened VIDS intervention.

Regardless of the fields being STEM or non-STEM (social science and humanities), the more raw talent it requires from individuals (e.g., brilliant and genie), the more men are represented within the field (Leslie et al., 2015). The authors further suggested expanding the study beyond STEM fields to gain more comprehensive solutions for gender disparities. Thus, we explored the possibility of an expanded VIDS intervention for gender bias by recruiting participants from both STEM and non-STEM fields. Furthermore, by recruiting Korean participants from the Korean society, the current research expands the range of audiences to non-Westerners in whom VIDS can be utilized.

The current study proposes two primary hypotheses. First, based on the previous research that shows watching VIDS significantly reduces gender bias (Moss-Racusin et al., 2018), we propose participants in the treatment group will report decreased gender bias after watching the shortened VIDS intervention, in contrast to the control group, with controlled cultural orientation (Hypothesis 1). Second, considering cognitive and affective perspective-taking engages different parts of the brain (Healey and Grossman, 2018), the treatment group will exhibit varied effectiveness in terms of increased emotional immersion and reinforced logical thinking from watching the narrative and expert video in reducing gender bias, respectively (Hypothesis 2). Hypotheses 1 and 2 were tested in a similar manner.

### Participants and recruitment

Participants were recruited from two Korean online communities: Blind and Kopas. Blind is an anonymous online community for verified employees to discuss issues online, mainly used by people in their 30s, whereas Kopas is an anonymous online community for Korea University students and faculty members, which has users majorly in their 20s (Kim et al., 2021).

Although we originally recruited 208 participants, 48 (23.1%) were excluded. Twenty-two (10.5%) participants incorrectly answered one or more (of six) simple attention check questions about the videos’ content, and 26 (12.5%) did not complete watching the videos. Thus, our final sample consisted of 160 Korean individuals in the age range of 19–39 years (*M* = 27.48, *SD* = 3.91), with 99 women (61.8%) and 61 men (38.2%). The current study originally aimed to evenly assign participants to the treatment and control groups; however, the final distribution of participants was 78 (48.8%) in the treatment group and 82 (51.2%) in the control group because of the exclusion of participants. Among the female participants, 47 (47.5%) and 52 (52.5%) were randomly assigned to the treatment group and the control group, respectively. Among the male participants, 31 (50.8%) and 30 (49.2%) were randomly assigned to the treatment and the control group, respectively. The participants had the following characteristics:40 (25%) high school diploma holders, 107 (66.9%) undergraduate degree holders, and 13 (8.1%) graduate degree holders; among the current and previous undergraduate and graduate school students, 51 (31.9%) and 109 (68.1%) majored in STEM and non-STEM fields, respectively. A 5,000 KRW (approximately 4.49 USD) beverage voucher was given as a reward to the participants for completing the experiment.

### Experiment design

The current study presented the video intervention to the participants then provided the questionnaires, meaning the participants only answered the questionnaires after watching the videos. Thus, the current experiment employed a post-test-only control group design, implying that gender bias questions were not presented as a pre-test but only as a post-test. Conducting a pre-test is desirable when the independent variable is expected to have a small effect, the number of participants is small, or the participants have diverse characteristics; otherwise, the experiment may result in a Type II error (Pasnak, 2018, p. 4473). The current experiment utilized an independent variable, VIDS, which was significant in decreasing gender bias in a previous study (Moss-Racusin et al., 2018), recruited a sufficient number of participants to hold power of 0.80 (Faul et al., 2009) and assessed gender bias through continuous and non-heterogeneous responses. Thus, the current study conducted an experiment without a pre-test to avoid Type II errors.

### Power analyses

The current study used G*Power 3.1 to perform power analysis to determine the minimum sample size required to test the study hypothesis (Faul et al., 2009). The analysis indicated that 128 participants were required to determine the effect of d = 0.50 (independent sample t-test, α = 0.05, power = 0.80). However, we recruited 208 participants owing to the possible exclusion of those who failed attention checks and who did not finish the videos. The final sample size of the current study (*n* = 160) surpassed the target of 128. Thus, the obtained sample size of *n* = 160 was adequate to test the study hypothesis.

### Video intervention for diversity in STEM

The VIDS is a series of intervention videos based on published research on gender bias in STEM fields (Pietri et al., 2017). It is the result of a collaboration of psychologists, biologists, professional playwrights, actors, and film producers and is accessible for academic use (Pietri et al., 2017). It consists of 12 videos under two conditions: narrative and expert. Six videos in the narrative condition are in TV show format with one main male or female character who experienced a gender bias in the STEM field. The six videos in the expert condition portray an interview between a psychology professor and an interviewer, who are professional actors, discussing academic findings and empirical evidence regarding gender bias. VIDS has been proven effective in decreasing gender bias in STEM fields through multiple experiments (Hennes et al., 2018; Moss-Racusin et al., 2018; Pietri et al., 2019).

A major advantage of employing VIDS is its provision of both narrative and expert videos with different working mechanisms. Previous research using VIDS found that narrative videos were more entertaining than expert videos, and that expert videos provided more information than narrative videos (Pietri et al., 2017). Plausible narratives can provide the necessary elements for perspective-taking, and the expertise shown in expert interviews helps to process information accurately and think critically (Boland and Tenkasi, 1995; Petty and Cacioppo, 1996). Thus, VIDS can be an effective perspective-taking tool for gender bias intervention, as narrative videos enable participants to emotionally engage with the main characters, and expert videos provide accurate information that promotes a logical understanding of the situation.

The present study employed VIDS based on the previous research of Moss-Racusin et al. (2018) to promote perspective-taking between male and female participants, and adapted the intervention time to sustain the participants’ attention. Based on research by The Ministry of Gender Equality and Family (2021), we selected the most suitable VIDS videos for promoting emotional immersion and logical thinking in both genders. To maximize the participants’ attention and completion of the videos, each video’s duration were less than 6 min and the entire intervention was less than 11 min (Guo et al., 2014; Geri et al., 2017). Regarding the content of the videos, the selection of the narrative and expert videos for male and female participants in the treatment group was based on research from The Ministry of Gender Equality and Family (2021). For the male participants the videos were selected based on that the younger generations of Korean men have less recognition of the socially expected gender roles that women are expected to play. For the woman participants in the treatment group, the videos were selected based on that in comparison to Korean men of the same generation, a lower percentage of Korean women acknowledged how society expects men to fulfill masculine gender roles. Each video was subtitled Korean.

### Video materials

#### Treatment group – male participants

##### VIDS narrative video A

The men in the treatment group were first asked to watch a narrative video. The video portrayed the subtle gender discrimination experienced by women in STEM fields. The script was based on published research on subtle gender biases in science faculty members and male student favoritism (Moss-Racusin et al., 2012). The male faculty member in the video, Kevin, assumes that Sarah, a female graduate student, does not know how to handle a microscope. Kevin seeks help from Peter, a male graduate student, rather than from Sarah, a laboratory technician, who believed that males in general possess more technical skills than females.

##### VIDS expert video A

The men in the treatment group watched an expert video. This video provides academic evidence of the subtle gender biases that women face in the STEM field. The video based on published research was in an interview format between an interviewer and a psychology professor, who were professional actors discussing gender bias. The professor and interviewer in the video introduced an experiment on subtle male favoritism in STEM fields and presented their results.

#### Treatment group – female participants

##### VIDS narrative video B

Women in the treatment group watched a narrative video based on published research on the gender roles society expects of men (Moss-Racusin et al., 2010). The video portrayed a subtle gender bias in two main characters in a TV show format. A male faculty member, Kevin, was unhappy with male graduate student Peter, who acted modestly. Kevin believed Peter should be more ambitious, competitive, and self-promoting. Towards the end, Kevin plans to cancel Peter’s travel grant because of his humble behavior.

##### VIDS expert video B

Women in the treatment group watched an expert video. This video presents the gender stereotypes within society that men experience in an academic context. The video script was based on previously published research (Moss-Racusin et al., 2010) in which an actor portrays a psychology professor who is a gender issue expert, and explains the backlash experienced by men for not conforming to the expected masculine roles in society.

#### Control group – male and female participants

##### Control video

A non-intervention video of a similar duration as the intervention videos was provided to the control group, regardless of gender. Participants in the control group were instructed to watch a Korean-subtitled control video about radium (Twig Education, 2019). This video explains how radium is used to treat cancer. Unlike VIDS videos, this video stimulus did not present any information on gender bias, even though it was presented to both male and female scientists, similar to the intervention videos.

### Attention checks

After watching the videos, all participants answered three questions based on the information provided in each video that they watched (e.g., “According to the video, what kind of feedback are humble men most likely to receive?”). This was to ensure participants’ adequate attention to the experiment and to exclude those who answered any of the questions incorrectly. We followed Moss-Racusin et al. (2018) attention check method. Four possible answers were presented to the participants with one correct answer (e.g., “a. Kind-hearted, b. Have low self-esteem, c. Menacing, d. Confident”). The participants in the treatment group answered six attention check questions.

### Emotional immersion and logical thinking

In addition to the attention check questions, the participants in the treatment group completed questionnaires assessing emotional immersion and logical thinking after watching each video. To measure emotional immersion and logical thinking, we utilized manipulation check questions from Moss-Racusin et al. (2018) that measured the two dimensions, respectively. Emotional immersion from the narrative video was measured using five items for assessing participants’ emotional involvement (e.g., “The narrative of the video affected me emotionally”; Green and Brock, 2000). However, one item (“While I was watching the video, the activity in the room was on my mind. [R]”) was excluded because it disturbed the internal consistency of the questionnaire. Thus, the final scale used in the analysis included four items. The answers ranged from 1 (strongly disagree) to 5 (strongly agree), with higher numbers indicating deeper emotional immersion (α = 0.77). Logical thinking was measured using two items assessing participants’ engagement in logical understanding of the situation (“The professor explained the point clearly with evidence and logic” and “The main argument of the video was supported by evidence and facts in a clear and logical manner”) ranging from 1 (strongly disagree) to 5 (strongly agree); responses with higher numbers reflected increased logical thinking (α = 0.95).

### Gender bias

Once the attention and manipulation check questions were completed by the treatment group, a gender stereotype scale was used to assess gender bias among the participants (Kim, 1993). The scale assesses gender stereotypes in Korean society with five subscales: domestic, traditional social role performance, vocational and physical, psychological, and intellectual gender bias (Kim, 1991). Seven items were used to examine domestic gender bias (e.g., “A man should be financially responsible for a household”), five items examined traditional social role performance gender bias (e.g., “Both men and women should hold similar authority within a family [R]”), 14 items examined vocational and physical gender bias (e.g., “Truck driver is not a good occupation for women,” “Physical appearance and youthfulness are more important for women than men”), seven items assessed psychological gender bias (e.g., “Men are more aggressive than women”), five items assessed intellectual gender bias (e.g., “In general, men perceive things more objectively than women”). Participants’ responses were rated on a scale of 1 (strongly disagree) to 5 (strongly agree) using 33 items on gender stereotypes in everyday life. The scores ranged from 33 to 165, with a higher score indicating a greater gender bias. We averaged the items to form a gender stereotypes scale. Cronbach’s α of the gender bias scale from a previous study by Kim and Kim (2019) was 0.89. The Cronbach’s α value in this study was obtained as 0.93.

### Cultural orientation

Cultural orientation was used as a control variable. We employed the individualism and collectivism scale (Triandis and Gelfand, 1998) to assess cultural orientation. The scale consists of 16 items assessing four dimensions of individualism and collectivism: Horizontal individualism (HI), horizontal collectivism (HC), vertical individualism (VI), and vertical collectivism (VC). Four items assessed horizontal individualism (e.g., “I’d rather depend on myself than others”) and four items examined horizontal collectivism (e.g., “If a coworker gets a prize, I will feel proud”). Four items assessed vertical individualism (e.g., “It is important that I do my job better than others”) and four items examined vertical collectivism (e.g., “It is my duty to take care of my family, even when I have to sacrifice what I want”). The Participants gave their responses on a scale of 1–9 (1 = definitely no, 9 = definitely yes). Each dimension includes four items on a scale with scores ranging from 4 to 36. A previous study that utilized the same scale (Coon and Kemmelmeier, 2001) reported the following Cronbach’s α for the various dimensions: HI (*α* = 0.68), HC (*α* = 0.63), VI (*α* = 0.82), and VC (*α* = 0.56). The following Cronbach’s α was reported in this study: HI (*α* = 0.81), HC (*α* = 0.76), VI (*α* = 0.65) and VC (*α* = 0.73).

### Demographic question

After completing all measures, the participants were asked to answer demographic questions on education and majors. The question on education had the following five choices: elementary, middle, high, undergraduate, and graduate school. The major question had eight possible answers: engineering, education, social sciences, art, medical studies, humanities, natural sciences, and none of these. The participants had the option to select multiple choices if they had multiple majors. Participants who majored in one or more courses in engineering, medical studies, and natural sciences were considered to have a STEM orientation. Participants who majored in education, social sciences, art, humanities, or none of the above were perceived as having a non-STEM orientation.

### Procedure

The research protocol of the current study was reviewed and approved by the Korea University Institutional Review Board in compliance with the standards for the ethical treatment of human participants before the data collection. The experiment was advertised in two anonymous online communities, Blind and Kopas, to explore the effect of watching videos on an individual’s gender bias. The participants had direct access to the experiment with a link provided through the advertisement. The online behavioral psychology experiment platform Gorilla was used to conduct the experiment, and participants were randomly assigned to treatment or control groups. Upon clicking on the experimental link, participants were first enquired about their age and provided with a brief explanation of the procedures and purpose of the experiment. Consistent with the purpose of the experiment, only participants aged 19–39 years were allowed to participate. Once the informed consent was provided by the participants, they were allocated to the treatment or control group through randomization.

Participants assigned to the treatment group answered a gender question first for their assignment to appropriate videos. Subsequently, the participants watched the intervention videos, followed by questions related to the video content. First, the participants watched a narrative video, followed by attention and manipulation check questions for the narrative video. Subsequently, they watched an expert video followed by attention and manipulation check questions for the expert video. It is significant that the perspective-taker should be consciously aware of the person they are trying to understand (Gehlbach and Mu, 2022), thus, the narrative video was shown prior to the expert video to present a specific target (e.g., the main actor of the narrative) to the participants. The participants in the treatment group later completed the gender bias and cultural orientation questionnaires. Finally, the demographic questions were answered and the participant’s submitted their email addresses to receive compensation.

Participants in the control group watched the irrelevant video on gender bias. They completed a gender bias questionnaire, cultural orientation questions, demographic questions, attention check questions, and manipulation check questions. Finally, they submitted an email address for compensation.

## Results

### Preliminary analysis

We have conducted descriptive statistics and bivariate correlation to assess if one’s cultural orientation is significantly correlated to gender bias. The results are presented in Table 1. As we anticipated, a significant negative correlation was found between gender bias and horizontal collectivism (*r* = −0.28, *p* < 0.001). No significant correlations with gender bias was observed for the remaining variables (horizontal individualism, vertical collectivism, and vertical individualism). This difference could be attributed to the fact that only horizontal collectivism is related to one’s favor toward altruism (Booysen et al., 2021), which motivates one to remain close and social with others (Shavitt et al., 2011). We have controlled the horizontal collectivism variable to examine hypothesis 1, to control the effect of cultural orientation on examining the effectiveness of the video intervention.

**Table 1:** Descriptive statistics and bivariate correlations between gender bias and cultural orientation variables.

Variables	1	2	3	4	5
1. Gender bias	–				
2. HI	−0.093	–			
3. HC	−0.279***	0.060	–		
4. VI	0.147	0.289***	0.091	–	
5. VC	0.102	0.017	0.359***	0.181*	–
M (SD)	1.97	7.23	6.48	6.37	6.20
M (SD)	(0.61)	(1.36)	(1.50)	(1.40)	(1.55)

HI, horizontal individualism; HC, horizontal collectivism; VI, vertical individualism; VC, vertical collectivism.

*p < 0.05, **p < 0.01, ***p < 0.001.

### Emotional immersion and logical thinking

Participants reported higher emotional immersion after watching the narrative video (*M* = 3.67, *SD* = 0.59) in comparison to the expert video (*M* = 3.36, *SD* = 0.61). Further, they reported higher logical thinking after watching the expert video (*M* = 4.09, *SD* = 0.97) compared to the narrative video (*M* = 3.17, *SD* = 1.04).

### Hypothesis testing

#### Hypothesis 1

We investigated the effectiveness of the shortened VIDS intervention in treatment and control groups. Using a one-way ANCOVAto control for horizontal collectivism, which had significant correlations with gender bias (*p* < 0.001), the efficacy of the VIDS intervention between the treatment and control groups was examined. Prior to conducting the one-way ANCOVA, we tested whether the horizontal collectivism variable met the equality of the within-group regression slopes. Table 2 presents the results of the analysis and the assumptions of the Levene’s test and normality checks (*p* = 0.078). The shortened VIDS intervention had a significant negative effect on gender bias after controlling for horizontal collectivism (*F* (1,157) =6.27, *p* < 0.05). The estimated marginal means were compared with those of the control group (*M* = 2.37) and gender bias decreased in the treatment group (*M* = 1.55).

**Table 2:** Analysis of covariance (ANCOVA) summary table for gender bias by group conditions.

Source	SS	*df*	MS	*F*	*p*-value
HC	1.68	1	1.68	4.60	0.034*
Group	2.29	1	2.29	6.27	0.013*
Error	57.42	157	0.37		
Total	680.25	160			

SS, sum of squares; MS, mean square; HC, horizontal collectivism.

*p < 0.05, **p < 0.01, ***p < 0.001.

#### Hypothesis 2

##### Assumption check

In our exploration of elements of perspective-taking on decreasing gender bias, we employed a multiple linear regression model to assess the contributions of predictor emotional immersion and logical thinking. Before examining the predictive power of our model, an assessment of its basic assumptions was analyzed to affirm the validity of our analysis. Homoscedasticity was confirmed via Breusch-Pagan test (*p* = 0.086). Furthermore, the Durbin-Watson statistics stood at 2.26, ruling out autocorrelation among residuals and attesting to the independence of errors. The Variance Inflation Factor (VIF) for each predictor was 1.104, below the threshold of 5, dispelling multicollinearity concerns. Collectively, these diagnostic tests validated the key assumptions of our multiple linear regression model.

Subsequently, multiple regression was run to test if the two dimensions of perspective-taking, emotional immersion and logical thinking, significantly predicted decreasing the level of gender bias. The results of the regression indicated the two predictors significantly explained 10% of the variance *F* (2, 75) = 5.287, *p* < 0.01, *R^2^* = 0.124, *R^2^*adjusted = 0.100. Emotional immersion added statistically significantly to the prediction, (*t* = −3.235, *p* < 0.01) while logical thinking did not (*t* = 0.682, *p* = 0.497). Table 3 shows the results of linear regression analysis.

**Table 3:** Linear regression analysis.

Variable	*B*	*SE*	95% CI		β	*t*	*p*-value
			LLCI	ULCI			
EI	−0.26	0.08	−0.427	−0.101	−0.37	−3.235**	0.002
LT	0.05	0.07	−0.091	0.186	0.08	0.682	0.497

EI, Emotional Immersion; LT, Logical Thinking.

*p < 0.05, **p < 0.01, ***p < 0.001.

**Figure 1:**
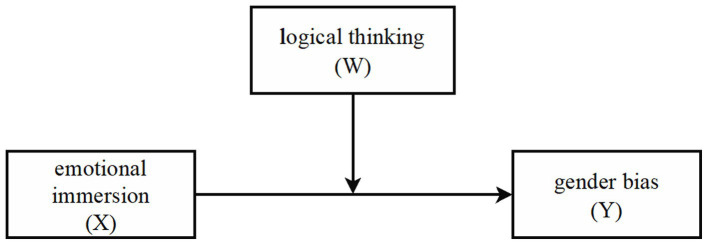
Moderation model of logical thinking in relationship between emotional immersion and gender bias.

Affection and cognition are inseparably intertwined and cognitive and emotional functioning are interconnected and cannot be performed in isolation (O’Rorke and Ortony, 1998; Pessoa, 2008). Consequently, a moderation analysis was conducted (Figure 1) to further investigate the mechanism between the two key elements of perspective taking (emotional immersion and logical thinking) and the reduction in gender bias. Through Hayes (2013) PROCESS macro Model 1, a statistical method used for moderation analysis in the context of regression analysis, we examined the moderating effect of logical thinking on emotional immersion and gender bias. Table 4 presents the results of moderation analyses. The results indicated that logical thinking moderated the relationship between emotional immersion and decreased gender bias (*p* < 0.05).

**Table 4:** Moderation analysis.

	*B*	*SE*	*t*	*p*-value	LLCI	ULCI
EI	−0.066	0.020	−3.327**	0.001	−0.105	−0.026
LT	0.099	0.071	1.395	0.167	−0.042	0.240
EI x LT	0.034	0.014	2.383*	0.020	0.006	0.062

EI, Emotional Immersion; LT, Logical Thinking.

*p < 0.05, **p < 0.01, ***p < 0.001.

**Figure 2:**
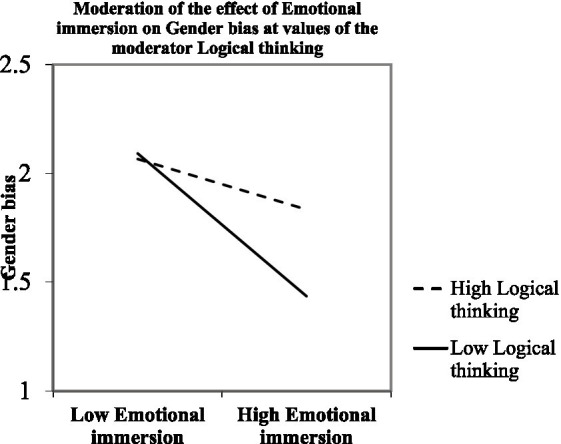
Moderation of the effect of emotional immersion on gender bias at values of the moderator logical thinking.

We employed a simple slope analysis, referred to as a pick-a-point approach (Montoya, 2019) to further examine how logical thinking moderates the relationship between emotional immersion and gender bias (Table 5). The results indicate that both −1 standard deviation (*B* = −0.099, *t* = −4.098, *p* < 0.001) and mean (*B* = −0.066, *t* = −3.327, *p* < 0.01) level of logical thinking had significant negative effects on the relationship between emotional immersion and gender bias, while +1 standard deviation (*B* = −0.035, *t* = −1.476, *p* = 0.144) level of logical thinking had an insignificant effect. Figure 2 illustrates the moderating effect of employing a pick-a-point approach. The results indicate that high logical thinking weakened the significant negative effects of emotional immersion on gender bias (Figure 2). Thus, participants who reported low to medium engagement in logical thinking after watching the expert video showed a significant decrease in gender bias from emotional immersion in the narrative video in comparison to participants who reported high engagement in logical thinking.

**Table 5:** Pick-a-point approach: moderation of logical thinking on the relationship between emotional immersion and gender bias.

Logical thinking	*B*	*SE*	*t*	*p*-value	LLCI	ULCI
−1SD	−0.099	0.024	−4.098***	0.000	−0.147	−0.051
M	−0.066	0.020	−3.327**	0.001	−0.105	−0.026
+1SD	−0.035	0.024	−1.476	0.144	−0.002	0.012

*p < 0.05, **p < 0.01, ***p < 0.001.

**Figure 3:**
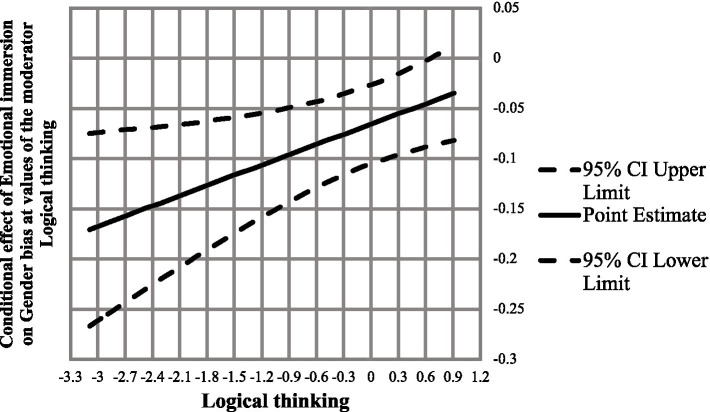
Conditional effect of emotional immersion on gender bias at values of the moderator logical thinking.

Since the interaction term was found to be significant, thus, the Johnson-Neyman technique was further employed to identify regions in the range of logical thinking where the effect of emotional immersion on decreasing gender bias is statistically significant and not significant. The result demonstrated that the effect of emotional immersion on gender bias had a significant negative effect within the range of-3.09 to 0.51 of logical thinking and an insignificant effect at 0.66 and above (Table 6; Figure 3).

**Table 6:** Conditional effect of logical thinking.

Logical thinking	Effect	*SE*	*t*	*p*-value	LLCI	ULCI
−3.09	−0.171	0.048	−3.543	0.001	−0.267	−0.075
−2.89	−0.164	0.046	−3.594	0.001	−0.255	−0.073
−2.69	−0.157	0.043	−3.649	0.000	−0.243	−0.071
−2.49	−0.150	0.041	−3.708	0.000	−0.231	−0.070
−2.29	−0.144	0.038	−3.769	0.000	−0.219	−0.068
−2.09	−0.137	0.036	−3.833	0.000	−0.208	−0.066
−1.89	−0.130	0.033	−3.897	0.000	−0.196	−0.064
−1.69	−0.123	0.031	−3.96	0.000	−0.185	−0.061
−1.49	−0.116	0.029	−4.019	0.000	−0.174	−0.059
−1.29	−0.110	0.027	−4.066	0.000	−0.163	−0.056
−1.09	−0.103	0.025	−4.095	0.000	−0.153	−0.053
−0.89	−0.096	0.023	−4.093	0.000	−0.143	−0.049
−0.69	−0.089	0.022	−4.045	0.000	−0.133	−0.045
−0.49	−0.082	0.021	−3.934	0.000	−0.124	−0.041
−0.29	−0.076	0.020	−3.746	0.000	−0.116	−0.035
−0.09	−0.069	0.020	−3.474	0.001	−0.108	−0.029
0.11	−0.062	0.020	−3.128	0.003	−0.102	−0.023
0.31	−0.055	0.020	−2.726	0.008	−0.096	−0.015
0.51	−0.049	0.021	−2.300	0.024	−0.091	−0.006
0.66	−0.044	0.022	−1.993	0.050	−0.087	0.000
0.71	−0.042	0.022	−1.876	0.065	−0.086	0.003
0.91	−0.035	0.024	−1.476	0.144	−0.082	0.012

## Discussion

The alleviation of the ongoing conflict over gender inequality in Korean society needs urgent addressal. The characteristics of gender conflict stem from differences in perceived discrimination among genders. Thus, it is crucial to understand the pre-existing gender stereotypes and biases which persist across generations. Therefore, effective interventions for reducing gender bias are significant. Hence, current study proposed to (a) examine whether watching brief videos of VIDS, which promote perspective taking, decrease gender bias, and (b) investigate the interaction of emotional and logical immersion on the reported reduction of gender bias.

Prior research revealing VIDS as an influential medium for decreasing gender bias (Moss-Racusin et al., 2018) was replicated and extended. Of the 12 VIDS videos, the current study provided one narrative and one expert video to the participants. The total intervention time approximated 10 min, which is almost 20 min lesser to that of previous research (Moss-Racusin et al., 2018). To minimize the impact of other factors for examining the efficacy of the shortened VIDS intervention, cultural orientation was controlled for due to its inclination for altruism which affects perspective-taking ability (Wu and Keysar, 2007; Booysen et al., 2021).

The results reported a significantly decreased gender bias in the treatment group compared with the control group (Hypothesis 1). These results indicate that briefly edited VIDS interventions may be effective in reducing gender bias which is similar to those of previous research. This could be attributed to the relationship between the intervention time and the participants’ sustained attention. Perspective-taking occurs when the cognitive and emotive information of others can be obtained, and adequate attention aids in efficient processing of information (Gehlbach, 2004; Pesimena et al., 2019). The current study promoted perspective-taking by providing logical and emotional information to the participants through the video intervention and optimized its efficiency as the videos’ duration was lesser than 6 minutes and further questionnaire on the story of the videos was provided, which sustained the participants’ attention to the maximum (Guo et al., 2014; Geri et al., 2017). Therefore, the shortened VIDS intervention may have contributed to maximizing the participants’ attention by providing them sufficient audiovisual cues to engage in effective perspective-taking. The findings indicate that briefly edited VIDS interventions can also be effective in decreasing gender bias, and suggest that VIDS interventions could be utilized in a time-saving manner.

This study first explored whether there is a difference between watching a narrative and an expert video in reducing gender bias, which increase emotional immersion and promote logical thinking, respectively (Hypothesis 2). The results showed that emotional immersion in the narrative video significantly reduced the gender bias. However, increased logical thinking had an insignificant impact in decreasing gender bias after watching the expert video. This finding contradicts the findings of Moss-Racusin et al. (2018) in which participants who watched six expert videos reported significantly lower gender bias, whereas participants who watched six narrative videos did not.

This difference in outcomes can be attributed to inter-individual variances in the participants. Both emotive and cognitive inter-individual functioning can be promoted or decreased differently depending on one’s state and traits (Stietz et al., 2019). Taken together, the findings of the current study suggest that people who mainly use emotional functioning to take others’ perspectives are better stimulated by presenting VIDS videos briefly, whereas those who use logical functioning when taking perspectives are better stimulated by longer VIDS stimulations. Thus, when utilizing VIDS interventions of different time durations, it is important to consider an individual’s affective and cognitive functioning. Perspective-taking largely depends on one’s situation, personality traits, mental health, and motivation to take perspective (Stietz et al., 2019). Therefore, none of the approach can be considered superior to the other, but both interventions are useful depending on the context or environment. This makes VIDS a more powerful perspective-taking tool, because various video conditions can be flexibly used under different circumstances.

In the current study, the differences in the participants’ sex distribution could also explain this contradiction. More women (62%) than men (38%) were included in the analysis due to the number of excluded participants. A previous study showed that men and women had different neural traits regarding gender biases and needed separate neural stimulators to restrain their implicit gender stereotypes (Wang et al., 2019, p. 9) Hence, in the current study, whose participants were shown fewer VIDS videos than Moss-Racusin et al. (2018) with more women in the sample, could have contributed to the differentiated result.

Additionally, the present study conducted a moderation analysis to investigate the interaction between increased emotional immersion and logical understanding in decreasing gender bias. The results reveal that logical thinking moderated the relationship between emotional immersion and gender biases. Specifically, the pick-a-point approach analysis showed that high logical thinking after emotional immersion eliminated the significant effect of emotional immersion on decreasing gender bias. When visual stimuli have low perceived relevance, rational appeals may fail to sustain viewers ‘attention and process the stimuli, whereas emotional appeal aids in maintaining attention to less relevant messages (Gong and Cummins, 2019). Since the participants in the current study watched videos that showed another gender’s experience of discrimination, not their own, the videos may have been perceived as less relevant to the participants. This may have resulted in the narrative video successfully sustaining the participants’ attention despite its low relevance, and engaging in high levels of logical thinking by watching the expert video immediately afterwards may have affected the sustained attention of the participants.

The findings of the current study have varied applications. Our results indicate that shortened VIDS exhibit varying levels of effectiveness in reducing gender bias depending on one’s emotional and cognitive understanding of another gender. We propose two main suggestions for utilizing shortened VIDS interventions based on the two key dimensions of perspective-taking, emotional immersion and logical thinking, considering that one’s inclination to take perspective is dependent on the situation and personal traits (Stietz et al., 2019).

Providing short videos in repetition that stimulate emotional engagement to individuals who mainly incorporate emotional immersion as a perspective-taking strategy could be effective since repetition is required due to people’s limited memory span (DeMarzo et al., 2003). Recently, content creators from short video-sharing platforms such as TikTok, YouTube, and Instagram have created numerous videos for entertainment and learning purposes (Zhang, 2020). With the platforms’ rapid content transmission, motivating content creators to produce short, emotionally engaging videos that can be used as gender-biased interventions by the general public may be effective (Zhang, 2020). Furthermore, inclusion of scenes that engage viewers emotionally with characters without gender bias or showing the characters taking perspective could also be influential, considering that viewers learn detailed interactions between characters from watching TV shows (Żerebecki et al., 2021, p. 8). Further, for emotive perspective takers promoting logical thinking immediately after increasing emotional immersion should be considered more carefully.

For people who use cognitive and logical functioning to take others’ perspectives, employing additional activities to develop a logical understanding of others’ situations can be helpful. Eyal et al. (2018) suggest that direct conversation is the most efficient way to increase the accuracy of social cognition. For instance, an interpersonal group which meets to directly obtain different genders’ cognitions of the world after watching shortened VIDS videos could be beneficial in reducing gender bias and encourage effective empathizing in logical perspective-takers.

This study not only confirms the effectiveness of shortened VIDS videos in reducing gender bias, but also has several implications for the expanded use of VIDS videos as interventions. First, it shows the possibility of utilizing the VIDS as a tool for perspective-taking. Previous research using VIDS as a gender bias intervention majorly examined the efficacy of videos consisting of narrative and expert conditions and did not promote perspective-taking between the participants. The increased level of emotive and logical engagement after watching the shortened VIDS suggests that VIDS can be utilized to promote understanding between genders. Since, genders perspective-taking using VIDS was effective in reducing gender bias among young adults in Korea, it can also be applied to other cultures that face similar issues. Second, this study expands the field in which the VIDS can be utilized. Although the intervention was originally designed to target STEM gender biases, the results of the current study showed its effectiveness for participants recruited from both STEM and non-STEM fields. Finally, previous studies employing VIDS have been majorly conducted in western countries (Pietri et al., 2017; Hennes et al., 2018; Moss-Racusin et al., 2018; Pietri et al., 2019). Employing the VIDS in Korean adults aged 19–39 years was effective in decreasing gender bias similar to the results of previous studies. This is the first study to use the VIDS in Korea, expanding the applicability of the VIDS in non-western cultures.

## Limitations and future directions

Future studies can further explore effective gender-biased interventions through numerous ways. First, the current study presented intervention videos in a fixed order to successfully initiate perspective taking (Gehlbach and Mu, 2022). Thus, all the participants in the treatment group watched the narrative video first, followed by the expert video. Prior research indicates that the order of information provided affects participants’ responses, and people form an impression based on the information provided first (Lasorsa, 2003; Sullivan, 2019). Thus, the order of the videos presented might have contributed to the narrative video showing more effectiveness in decreasing gender bias than expert videos. Future studies can explore the impact of presenting the expert video first or randomizing the order of the videos on perspective-taking and investigate its effect in decreasing gender bias.

Furthermore, perspective taking can be performed more accurately if there is a direct interaction between groups (Eyal et al., 2018). Thus, to promote more accurate perspective-taking, in addition to presenting VIDS, future studies should include direct interactions between genders.

The current study attempted to recruit non-binary participants as suggested by Farrell et al. (2020) in their study on perspective taking affecting gender biases. This can assist in generalization of VIDS results to more diverse populations. None of the participants reported their gender orientation as non-binary. Therefore, our study only investigated the effects of short VIDS interventions in a binary population. Future studies could attempt to recruit individuals who identify as non-binary to examine the applicability of these findings to more diverse participants. Likewise, future studies should explore how the promotion of perspective taking in VIDS videos can be effective for non-binary participants.

Another possible consideration is the cultural difference between the stimuli (VIDS) and participants (South Koreans) in this study. The VIDS was invented in predominantly individualistic western cultures, whereas the participants in the current study were from East Asia, where the culture is mainly collectivistic (Wu and Keysar, 2007). The current study assessed and controlled for the effect of horizontal cultural orientation, which is associated with collectivistic Korean culture and the inclination to empathize (Lee et al., 2007; Shavitt et al., 2011; MacNeill and Wozniak, 2018; Booysen et al., 2021). Future studies can explore whether VIDS can show effectiveness without controlling for the effect of cultural orientation or if there is a way of utilizing a more culturally sensitive VIDS.

Finally, the current study focused on the population in the age range of 19–39 years, which is experiencing maximum gender disparities. Since our results revealed that a short intervention using the VIDS could be effective for people aged 19–39 years, future research can explore the adaptation of the same intervention in older or even younger generations.

## Conclusion

The finding of the study that perspective-taking through a shortened VIDS intervention can help combat personal gender bias is noteworthy. Our results show the efficient use of VIDS even under time constraints. Additionally, our findings indicate that the increased emotional immersion and encouraged logical thinking after watching short VIDS videos have varying impact in decreasing gender bias. Furthermore, moderation analysis showed that the depth of logical thinking significantly affected emotional immersion in reducing gender bias. This highlights the importance of considering individuals’ perspective-taking strategies for effective implementation of VIDS interventions. This is the first study to employ the VIDS in Korea, depicting the possibility of its adoption in Korean society. As reducing gender bias is critical for combating gender discrimination that is persistent across generations, this study presents implications for gender equity.

## Areas of application

To narrow gender gaps, especially in male-dominated areas, it is crucial to offer easily accessible education for women as well as prevent early leavers, which are an important dimension in educating youth (Gaweł and Krstić, 2021). The findings of our study suggest utilizing briefly edited VIDS could be an approachable intervention to the viewers and be an educational tool for gender equity. In class, students can watch the shortened intervention videos and engage in activities to take the perspectives of other genders through various forms such as writing, presentations, etc. Having a discussion session between various genders to share their thoughts after watching the videos can also be beneficial on decreasing bias, since obtaining new and correct information from others is important in accurate perspective-taking (Eyal et al., 2018). It is also possible for students to create their own short-form intervention videos as a project, based on their life experiences of biased treatment, considering having high perceived relevance makes rational and emotional appeal more effective, especially in sustaining viewers’ attention (Gong and Cummins, 2019). Furthermore, watching the videos together can serve as an informative tool, such as raising the viewers’ awareness of existing gender bias or inspiring them to seek ways to minimize repeating such bias. As creating videos is a popular medium for the younger generation to communicate, utilizing short video interventions, can be an effective strategy to decrease gender bias.

## Data availability statement

The original contributions presented in the study are included in the article/Supplementary material, further inquiries can be directed to the corresponding author.

## Ethics statement

The studies involving humans were approved by Korea University Institutional Review Board (IRB-2021-0266). The studies were conducted in accordance with the local legislation and institutional requirements. The participants provided their written informed consent to participate in this study.

## Author contributions

YB: Writing – original draft. JJ: Writing – review & editing.
