# Supplementary material for: The effectiveness of a brief video-based intervention in reducing gender bias in Korea
Source: Front Psychol. 2024 Apr 9;15:1331460. doi: 10.3389/fpsyg.2024.1331460 (PMC11037398; doi:10.3389/fpsyg.2024.1331460)
Supplement: Supplementary file 1 [file Image_1.pdf]

Emotional Immersion Questionnaire (Green & Brock, 2000; Moss-Racusin et al., 2018)

1. While I was reading the narrative, activity going on in the room around me was on my mind.

1	2	3	4	5
strongly disagree				strongly agree

2. I was mentally involved in the narrative while reading it.

1	2	3	4	5
strongly disagree				strongly agree

3. I wanted to learn how the narrative ended.

1	2	3	4	5
strongly disagree				strongly agree

4. The narrative affected me emotionally.

1	2	3	4	5
strongly disagree				strongly agree

5. The events in the narrative are relevant to my everyday life.

1	2	3	4	5
strongly disagree				strongly agree
