# Supplementary material for: The effectiveness of a brief video-based intervention in reducing gender bias in Korea
Source: Front Psychol. 2024 Apr 9;15:1331460. doi: 10.3389/fpsyg.2024.1331460 (PMC11037398; doi:10.3389/fpsyg.2024.1331460)
Supplement: Supplementary file 2 [file Image_2.pdf]

Individualism and Collectivism Scale (Triandis & Gelfand, 1998)

Horizontal individualism items

1. I'd rather depend on myself than others.

1	2	3	4	5	6	7	8	9
never								always

2. I rely on myself most of the time; I rarely rely on others.

1	2	3	4	5	6	7	8	9
never								always

3. I often do "my own thing."

1	2	3	4	5	6	7	8	9
never								always

4. My personal identity, independent of others, is very important to me.

1	2	3	4	5	6	7	8	9
never								always

Vertical individualism items

1. It is important that I do my job better than others.

1	2	3	4	5	6	7	8	9
never								always

2. Winning is everything.

1	2	3	4	5	6	7	8	9
never								always

3. Competition is the law of nature

1	2	3	4	5	6	7	8	9
never								always

4. When another person does better than I do, I get tense and aroused.

1	2	3	4	5	6	7	8	9
never								always

Horizontal collectivism items

1. If a coworker gets a prize, I would feel proud.

1 2 3 4 5 6 7 8 9
never always

2. The well-being of my coworkers is important to me

1 2 3 4 5 6 7 8 9
never always

3. To me, pleasure is spending time with others

1 2 3 4 5 6 7 8 9
never always

4. I feel good when I cooperate with others.

1 2 3 4 5 6 7 8 9
never always

Vertical collectivism items

1. Parents and children must stay together as much as possible.

1 2 3 4 5 6 7 8 9
never always

2. It is my duty to take care of my family, even when 1 have to sacrifice what I want.

1 2 3 4 5 6 7 8 9
never always

3. Family members should stick together, no matter what sacrifices are required.

1 2 3 4 5 6 7 8 9
never always

4. It is important to me that I respect the decisions made by my groups.

1 2 3 4 5 6 7 8 9
never always
