# Supplementary material for: The effectiveness of a brief video-based intervention in reducing gender bias in Korea
Source: Front Psychol. 2024 Apr 9;15:1331460. doi: 10.3389/fpsyg.2024.1331460 (PMC11037398; doi:10.3389/fpsyg.2024.1331460)
Supplement: Supplementary file 3 [file Image_3.pdf]

Korean Gender Bias Scale (Kim, 1993)

1. It's better for mothers with kids to stay home.
1 2 3 4 5
strongly disagree strongly agree

2. It's nice for women to take care of the home and family while men work outside the home.
1 2 3 4 5
strongly disagree strongly agree

3. A man should be financially responsible for a household.
1 2 3 4 5
strongly disagree strongly agree

4. It's better for women to succeed by assisting her spouse's career development, rather than developing her own.
1 2 3 4 5
strongly disagree strongly agree

5. Women should be first responsible for taking care of the home even if she has a job.
1 2 3 4 5
strongly disagree strongly agree

6. If one of the husband and the wife has to resign from the job, it should be the wife.
1 2 3 4 5
strongly disagree strongly agree

7. It's nice for women to take care of the home and family while men work outside the home.
1 2 3 4 5
strongly disagree strongly agree

8. Physical appearance and youthfulness are more important for women than men.
1 2 3 4 5
strongly disagree strongly agree

9. Nurse is a more suitable job for women than men.
1 2 3 4 5
strongly disagree strongly agree

10. It is important for women to look attractive.
1 2 3 4 5
strongly disagree strongly agree

11. Switchboard operater is more suitable job for women than men.
1 2 3 4 5
strongly disagree strongly agree

12. It is more suitable for women to work as servers at a restaurant.
1 2 3 4 5
strongly disagree strongly agree

13. Skirts on women looks better than pants.
1 2 3 4 5
strongly disagree strongly agree

14. Women take more care about their appearance than men.
1 2 3 4 5
strongly disagree strongly agree

15. Truck driver is not a good occupation for women.
1 2 3 4 5
strongly disagree strongly agree

16. Men suit more as a head of an institution than women.
1 2 3 4 5
strongly disagree strongly agree

17. It is desirable for women to have their own career.
1 2 3 4 5
strongly disagree strongly agree

18. Both men and women should hold similar authority within a family.
1 2 3 4 5
strongly disagree strongly agree

19. Both men and women should be given equal political rights.
1 2 3 4 5
strongly disagree strongly agree

20. It is an outdated thought that a family must have a boy.
1 2 3 4 5
strongly disagree strongly agree

21. It is not desirable for women with careers to have or raise a baby.
1 2 3 4 5
strongly disagree strongly agree

22. Women are less authoritative than men.
1 2 3 4 5
strongly disagree strongly agree

23. Women are less assertive than men.

1	2	3	4	5
strongly disagree				strongly agree

24. Women are less adventurous than men.

1	2	3	4	5
strongly disagree				strongly agree

25. Men are more aggressive than women.

1	2	3	4	5
strongly disagree				strongly agree

26. Women are less ambitious than men.

1	2	3	4	5
strongly disagree				strongly agree

27. Women has less leadership than men.

1	2	3	4	5
strongly disagree				strongly agree

28. Women are submissive compared to men.

1	2	3	4	5
strongly disagree				strongly agree

29. In general, men perceive things more objectively than women.

1	2	3	4	5
strongly disagree				strongly agree

30. Men are more creative than women.

1	2	3	4	5
strongly disagree				strongly agree

31. Men are more competent than women overall.

1	2	3	4	5
strongly disagree				strongly agree

32 Men are more rational than women.

1	2	3	4	5
strongly disagree				strongly agree

33. Men are better at math than women.

1	2	3	4	5
strongly disagree				strongly agree
