# Supplementary material for: The effectiveness of a brief video-based intervention in reducing gender bias in Korea
Source: Front Psychol. 2024 Apr 9;15:1331460. doi: 10.3389/fpsyg.2024.1331460 (PMC11037398; doi:10.3389/fpsyg.2024.1331460)
Supplement: Supplementary file 4 [file Image_4.pdf]

Logical Thinking Questionnaire (Moss-Racusin et al., 2018)

1. The professor explained the point clearly with evidence and logic

1	2	3	4	5
strongly disagree				strongly agree

2. The main argument of the video was supported by evidence and facts in a clear and logical manner

1	2	3	4	5
strongly disagree				strongly agree
